# Incidence, Risk, and Severity of SARS-CoV-2 Reinfections in Children and Adolescents Between March 2020 and July 2022 in Serbia

**DOI:** 10.1001/jamanetworkopen.2022.55779

**Published:** 2023-02-13

**Authors:** Snežana Medić, Cleo Anastassopoulou, Zagorka Lozanov-Crvenković, Nataša Dragnić, Vladimir Petrović, Mioljub Ristić, Tatjana Pustahija, Athanasios Tsakris, John P. A. Ioannidis

**Affiliations:** 1 Department of Epidemiology, Faculty of Medicine, University of Novi Sad, Novi Sad, Serbia; 2 Center for Disease Control and Prevention, Institute of Public Health of Vojvodina, Novi Sad, Serbia; 3 Department of Microbiology, Medical School, National and Kapodistrian University of Athens, Athens, Greece; 4 Department of Mathematics and Informatics, Faculty of Science, University of Novi Sad, Novi Sad, Serbia; 5 Department of Social Medicine and Health Statistics With Informatics, Faculty of Medicine, University of Novi Sad, Novi Sad, Serbia; 6 Center for Informatics and Biostatistics, Institute of Public Health of Vojvodina, Novi Sad, Serbia; 7 Department of Medicine, Stanford University, Stanford, California; 8 Department of Epidemiology and Population Health, Stanford University, Stanford, California; 9 Department of Biomedical Data Science, Stanford University, Stanford, California; 10 Department of Statistics, Stanford University, Stanford, California

## Abstract

**Importance:**

During the COVID-19 pandemic, children and adolescents were massively infected worldwide. In 2022, reinfections became a main feature of the endemic phase of SARS-CoV-2, so it is important to understand the epidemiology and clinical impact of reinfections.

**Objective:**

To assess the incidence, risk, and severity of pediatric SARS-CoV-2 reinfection.

**Design, Setting, and Participants:**

This retrospective cohort study used epidemiologic data of documented SARS-CoV-2 infections from the surveillance database of the Institute for Public Health of Vojvodina. A total of 32 524 children and adolescents from Vojvodina, Serbia, with laboratory-confirmed SARS-CoV-2 infection between March 6, 2020, and April 30, 2022, were followed up for reinfection until July 31, 2022.

**Main Outcomes and Measures:**

Incidence rates of documented SARS-CoV-2 reinfection per 1000 person-months, estimated risk of documented reinfection 90 days or more after laboratory confirmation of primary infection, reinfection severity, hospitalizations, and deaths.

**Results:**

The study cohort included 32 524 children and adolescents with COVID-19 (mean [SD] age, 11.2 [4.9] years; 15 953 [49.1%] male), including 964 children (3.0%) who experienced documented reinfection. The incidence rate of documented reinfections was 3.2 (95% CI, 3.0-3.4) cases per 1000 person-months and was highest in adolescents aged 12 to 17 years (3.4; 95% CI, 3.2-3.7). Most reinfections (905 [93.9%]) were recorded in 2022. The cumulative reinfection risk was 1.3% at 6 months, 1.9% at 9 months, 4.0% at 12 months, 6.7% at 15 months, 7.2% at 18 months, and 7.9% after 21 months. Pediatric COVID-19 cases were generally mild. The proportion of severe clinical forms decreased from 14 (1.4%) in initial episodes to 3 (0.3%) in reinfections. Reinfected children were approximately 5 times less likely to have severe disease during reinfection compared with initial infection (McNemar odds ratio, 0.2; 95% CI, 0.0-0.8). Pediatric reinfections rarely led to hospitalization (0.5% vs 1.3% during primary infections), and none resulted in death.

**Conclusions and Relevance:**

This cohort study found that the SARS-CoV-2 reinfection risk remained substantially lower for children and adolescents compared with adults as of July 2022. Pediatric infections were mild, and reinfections were even milder than primary infections.

## Key Points

**Question:**

What are the incidence, risk, and severity of SARS-CoV-2 reinfection in children and adolescents?

**Findings:**

This cohort study followed up 32 524 children and adolescents with primary SARS-CoV-2 infections in Vojvodina, Serbia, for reinfections, and found that the risk of pediatric reinfection remained less than 8% approximately 2 years into the pandemic, with an incidence rate of 3.2 cases per 1000 person-months. Pediatric COVID-19 cases were generally mild, and reinfections were even milder.

**Meaning:**

These findings suggest that documented reinfection risk remains substantially lower in the pediatric vs adult population, with an even more favorable profile compared with primary infections.

## Teaser

This cohort study of children and adolescents in Vojvodina, Serbia, assesses the reinfection risk of SARS-CoV-2.

## Introduction

Age is an important parameter affecting the clinical outcome of SARS-CoV-2 infections.^1^ In most children and adolescents, infections are mild or asymptomatic, although severe illness, including a rare multisystem inflammatory syndrome, has been observed, particularly among young children.^2,3^ Despite the perhaps inevitable underreporting of subclinical infections, surveillance data from the pediatric population provided real-time information on symptomatic infections during the COVID-19 pandemic, informing risk assessments of the spread of the virus in schools.^4,5^

Data on SARS-CoV-2 reinfection in pediatric cohorts are scarce and mainly cover the pre-Omicron period. Studies have found a lower reinfection risk for children compared with adults and no association with more severe disease or fatal outcomes.^6,7^ A surveillance study^6^ from England concluded that reinfection rates followed community infection rates. Symptomatic reinfections represented a large proportion of symptomatic COVID-19 cases (19.8%) among children in Nicaragua.^2^ Overall, the risk and severity of reinfection in children remain poorly characterized.

We aimed to assess the incidence, severity, and risk of SARS-CoV-2 reinfection in children and adolescents by analyzing surveillance data for the Autonomous Province of Vojvodina, Serbia, from the beginning of the pandemic (March 6, 2020) to the end of July 2022. We hypothesized that reinfections would be less severe than primary infections and that the probability of reinfection varies depending on the pandemic wave in which the primary infection occurred.

## Methods

### Study Design

We conducted a retrospective, population-level cohort study to assess the incidence, risk, and severity of SARS-CoV-2 reinfections in children and adolescents younger than 18 years in Vojvodina, Serbia, with primary infections documented between March 6, 2020, and April 30, 2022. Reinfections were monitored until the end of follow-up (July 31, 2022) or date of death. Primary or first infection was defined as detection of SARS-CoV-2 RNA or antigen in nasopharyngeal swab samples. Reinfection was defined based on a positive reverse transcription–polymerase chain reaction (RT-PCR) or rapid antigen test (Ag-RDT) result at least 90 days after laboratory confirmation of primary infection.^6,8^ The collection of samples for COVID-19 diagnosis formed part of the standard patient management in the frame of public health surveillance. Oral informed consent was obtained from patients (or legal guardians of children younger than 16 years). Patients’ data were anonymized before analysis. Data were accessible only to authorized employees of the Institute for Public Health of Vojvodina (IPHV). Ethical approval for this study was obtained from the ethics committee of IPHV. The study followed the Strengthening the Reporting of Observational Studies in Epidemiology (STROBE) reporting guideline.^9^

The study was conducted at IPHV, which provides oversight of the surveillance of infectious diseases among approximately 1.9 million inhabitants of Vojvodina (26.7% of the Serbian population).^10^ The surveillance IPHV database contains data for Vojvodina residents with laboratory-confirmed SARS-CoV-2 from March 6, 2020. COVID-19 surveillance is case based and fully covers the population of Vojvodina. In the early phases of the COVID-19 pandemic, movement in and out of the province was limited because of imposed lockdowns. Travel remained restricted in 2021.^11^ Data originated from epidemiologic questionnaires and included information on patients’ sex, age, residence, symptoms, date of symptom onset, illness severity, number of comorbidities, hospitalization, date of SARS-CoV-2 laboratory confirmation, diagnostic test used, date of case registration, and disease outcome (recovery or death within 90 days of testing positive). The cause of death was classified as COVID-19 or non–COVID-19, according to the patients' death certificates.

For surveillance purposes, COVID-19 severity was classified as mild if no pneumonia was diagnosed by chest imaging, severe if COVID-19 pneumonia was confirmed by chest imaging, and critical if illness required admission to the intensive care unit and/or mechanical ventilation. A Unique Master Citizen identification number was used to search the surveillance database for documented reinfections and to link recorded cases with the national registry of administered COVID-19 vaccines. Obtained data on vaccination status were automatically updated during follow-up and included the dates, dose number, and type of vaccine administered. For 4.8% of cases for which the identification number was missing, the search was performed by patient name, surname, date of birth, and residence. A total of 33 782 cases of COVID-19 among children and adolescents were registered in the database. Overall, 1258 primary infections, registered between April 30 and July 31, 2022, were excluded because of the limited time for reinfection to occur (according to the ≥90 days case definition). The distribution of SARS-CoV-2 variants in Europe during pandemic waves is shown in eAppendix in Supplement 1. B.1.1.7 (Alpha) and B.1.617.2 (Delta) were the most important variants epidemiologically and clinically (variants of concern) to this date.^12^

### COVID-19 Diagnostic Algorithm

The diagnostic algorithm has been previously described.^13^ In brief, laboratory-confirmed cases were defined based on positive RT-PCR or Ag-RDT results of posterior nasopharyngeal swab samples within the first 5 days of illness. Patients with COVID-19 symptoms and negative Ag-RDT results were tested by RT-PCR in repeated nasopharyngeal swabs.

### Classification of Cases by Vaccination Status

In Serbia, vaccination against COVID-19 (with messenger RNA vaccines only) has been available since February 2021 for adolescents aged 16 to 17 years and since June 2021 for children 12 years or older, with a booster dose introduced in March 2022. Vaccination status (unvaccinated, partially or fully vaccinated, and boosted) was defined according to the number of received vaccine doses in relation to the date of appearance of reinfection symptoms (or a positive test result in asymptomatic cases).

### Statistical Analysis

The share of documented primary SARS-CoV-2 infections and reinfections in the pediatric population was examined as proportions. Descriptive statistics of demographic characteristics of participants were generated. The following age groups were analyzed: 0 to 1, 2 to 11, and 12 to 17 years. Examined variables included the presence of comorbidities and COVID-19 severity, including hospitalization. The proportions of primary infection that were reinfected was assessed by the Cochran-Armitage test. Paired McNemar tests were used to compare reinfection vs primary infection in analyses with severe disease and hospitalization outcomes.

Incidence rates of reinfection were expressed per 1000 person-months. Age-specific incidence of primary infection and reinfection per 100 000 inhabitants was calculated using the 2021 population estimates.^10^ The mean period between primary infection and reinfection was calculated. Time-to-event Kaplan-Meier analysis was used to estimate the reinfection risk during follow-up, starting at 3 months after primary infection. We also estimated the risk of severe or critical disease over time. COVID-19 vaccine uptake in the pediatric cohort was followed from the middle of February 2021 until July 31, 2022. Stata, version 16 (StataCorp LLC) and TIBCO Statistica, version 14.0.0 (Cloud Software Group) were used.

## Results

### Primary SARS-CoV-2 Infections and Reinfections in the Pediatric Population

**Figure 1.**
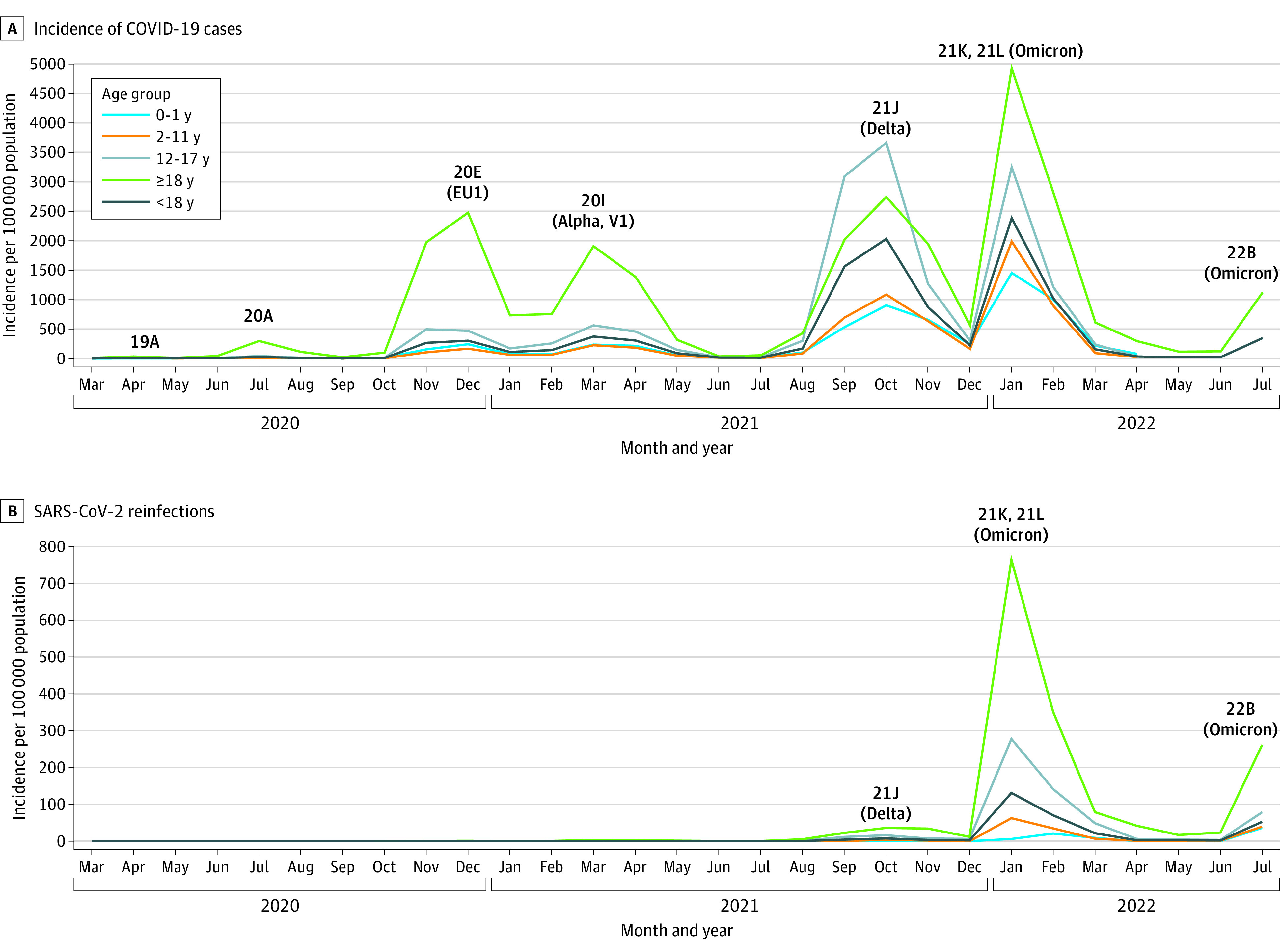
Age-Specific Incidences of Primary SARS-CoV-2 Infections and SARS-CoV-2 Reinfections in the Pediatric and Adult Populations of Vojvodina, Serbia, March 6, 2020, to July 31, 2022

The cohort included 32 524 of 33 782 COVID-19 cases registered in children and adolescents (mean [SD] age, 11.2 [4.9] years; 15 953 [49.1%] male and 16 571 [50.9%] female) in the IPHV database from March 6, 2020, until April 30, 2022 (eTables 1 and 2 in Supplement 1). Increases were noted in 2021 in the recorded share of pediatric cases (from 2047 of 78 845 [2.6%] in 2020 to 18 967 of 213 645 [8.9%] in 2021), with surges in September (5015 of 18 967 [26.4%]) and October (6506 of 18 967 [34.3%]) (eTable 1 in Supplement 1; Figure 1A). In 2022, pediatric cases remained at similar levels (12 768 of 163 844 [7.8%]), with most infections (7638 of 12 768 [59.8%]) recorded in January 2022.

Of the 32 524 documented pediatric primary infections until April 30, 2022, a total of 964 patients (3.0%) experienced reinfection until July 31, 2022 (eTable 2 in Supplement 1). The mean (SD) time from primary infection to reinfection was 240 (117) days (range, 90-591 days). Most infections were confirmed by Ag-RDTs (30 388 of 32 524 [93.4%]). No reinfections were documented in the pediatric cohort in 2020, few (59 of 964 [6.1%]) in 2021, and most (905 of 964 [93.9%]) in 2022 (eTable 2 in Supplement 1). The highest proportion of primary infections with reinfections was recorded in June 2021 (7 of 55 [12.7%]) during the fourth pandemic wave, with decreasing trends afterward (eTable 2 and eFigure 1 in Supplement 1).

### Incidence and Estimated Risk of Reinfections in Children and Adolescents

Similar incidence patterns of primary infection and reinfection were noted in the pediatric vs the adult population but to a lower extent in the former (Figure 1B). In adults, reinfections surged during the sixth (January 1 to June 30, 2022) and seventh (July 2022 and later) pandemic waves, when the 21K and 22B Omicron subvariants, respectively, predominated, with peaks in January and July 2022 (766.6 and 262.1 cases per 100 000 inhabitants, respectively). This reinfection profile was mirrored in children and adolescents but at an approximately 5.5-fold lower incidence. Pediatric reinfections also peaked in January 2022 (131.4 per 100 000 population). The highest incidence, recorded in 12- to 17-year-old adolescents, was 4.4-fold higher than the incidence in children aged 2 to 11 years (278.3 and 62.6 per 100 000 population, respectively). Both primary infection and reinfection rates were lower in younger (<12 years) pediatric age groups compared with older children (Figure 1).

The overall incidence rate of documented pediatric reinfections was 3.2 (95% CI, 3.0-3.4) cases per 1000 person-months (Table). Rates did not differ much by sex, age, comorbidities, severity of primary infection, or vaccination status. The highest rate was recorded in adolescents aged 12 to 17 years (3.4; 95% CI, 3.2-3.7). The mean (SD) age among participants with reinfections was 12.5 (4.3) years (vs 11.1 [4.9] years among those without reinfection).

**Table.** Incidence Rates of SARS-CoV-2 Reinfection in Children and Adolescents in Vojvodina, Serbia, March 6, 2020, to July 31, 2022

Characteristic	No. (%) of COVID-19 cases (N = 32 524)	No. (%) of reinfections (n = 964)	Total No. of person-months (N = 299 591)	Incidence rate of reinfections per 1000 person-months (95% CI)	Incidence rate ratio (95% CI)
Sex					
Male	15 953 (49.1)	499 (51.8)	151 611	3.3 (3.0-3.6)	1.1 (0.9-1.2)
Female	16 571 (50.9)	465 (48.2)	147 980	3.1 (2.9-3.4)	1 [Reference]
Age group, y					
0-1	2172 (6.7)	53 (5.5)	19 490	2.7 (2.0-3.6)	1 [Reference]
2-11	11 920 (36.6)	305 (31.6)	103 483	2.9 (2.6-3.3)	1.1 (0.8-1.5)
12-17	18 432 (56.7)	606 (62.9)	176 618	3.4 (3.2-3.7)	1.3 (0.9-1.7)
Comorbidities^a^					
0	31 818 (97.8)	936 (97.1)	291 903	3.2 (3.0-3.4)	1 [Reference]
≥1	706 (2.2)	28 (2.9)	7688	3.8 (2.5-5.4)	1.1 (0.8-1.7)
Severity of disease^b^					
Mild	32 182 (98.9)	950 (98.5)	293 629	3.2 (3.0-3.5)	1.4 (0.8-2.5)
Severe	342 (1.1)	14 (1.5)	5962	2.4 (1.3-3.9)	1 [Reference]
Vaccination status^c^					
Unvaccinated	32 315 (99.4)	963 (99.9)	298 197	3.2 (3.0-3.4)	1.3 (0.2-52.9)
Partially	58 (0.1)	1 (0.1)	414	2.4 (0.0-13.5)	1 [Reference]
Fully vaccinated plus boosted	151 (0.5)	0	980	0	0

^a^ Comorbidities included hypertension, diabetes, chronic pulmonary disease, chronic cardiovascular disease, malignancy, obesity, and other chronic diseases.

^b^ There were no critical cases of COVID-19 in the pediatric population.

^c^ At the time of laboratory confirmation of the primary infection, using the following definitions. Unvaccinated included those who had received 1 dose at 14 days or earlier before the date of reinfection symptoms onset (or positive test result in asymptomatic cases). Partially vaccinated included those who had received 1 dose and more than 14 days had passed since the vaccination day or the vaccination schedule had not been completed or if the final dose was given 14 days or earlier or more than 6 months before the reinfection date. Fully vaccinated included those who had received 2 vaccine doses and the final dose was received more than 14 days and 6 months or less before the reinfection date. Boosted included those in whom more than 7 days had passed since receiving the third (booster) dose before the reinfection date.

**Figure 2.**
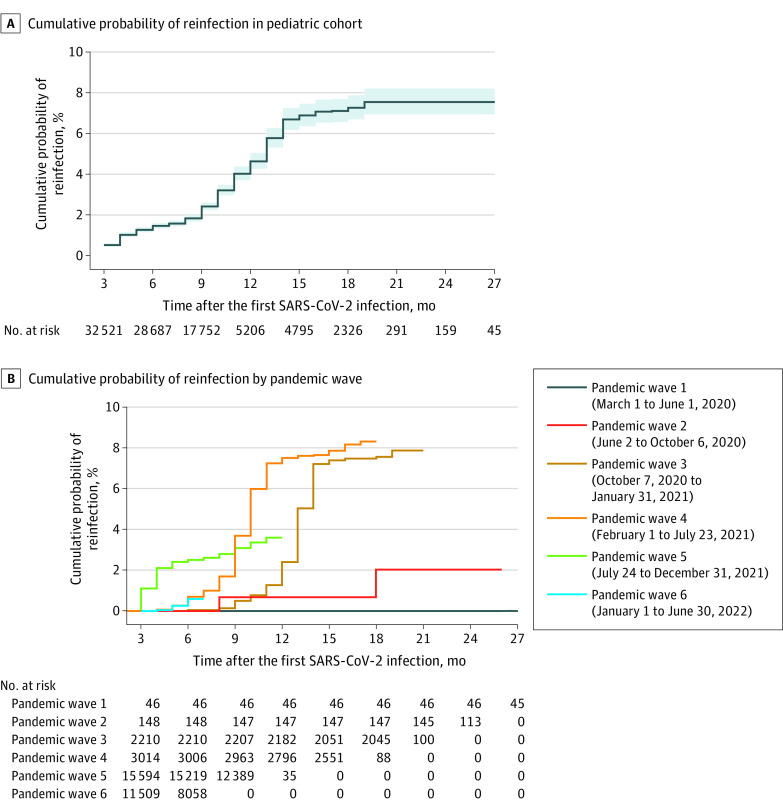
Kaplan-Meier Curves Showing the Cumulative Probability of Reinfection in the Pediatric Cohort and According to Pandemic Waves in Vojvodina, Serbia, March 6, 2020, to July 31, 2022

Figure 2A shows the Kaplan-Meier plot for the cumulative reinfection risk for pediatric patients who survived 3 months after primary infection (n = 32 521). The risk becomes 1.3% at 6 months, 1.9% at 9 months, 4.0% at 12 months, 6.7% at 15 months, and 7.2% at 18 months and remains the same after 21 months (7.9%) to the end of follow-up. The probability of reinfection was highest for children and adolescents who experienced primary infection during the third (October 7, 2020, to January 31, 2021) and fourth (February 1 to July 23, 2021) pandemic waves, presumably with the predominating variants at the time, 20I (Alpha, V1) and 21J (Delta), respectively (Figure 2B). The risk of reinfection did not differ substantially for patients with mild vs severe primary infections (eFigure 2A in Supplement 1), in different age groups (eFigure 2B in Supplement 1), or between the sexes (eFigure 2C in Supplement 1).

### Severity of Pediatric Reinfections Compared With Primary Infections

Pediatric COVID-19 cases were generally mild (Table; eFigure 3 in Supplement 1). The share of severe clinical forms decreased from 14 (1.4%) in initial episodes to 3 (0.3%) in reinfections. Reinfected patients were 4.7 times less likely to have severe disease during reinfection compared with initial infection (McNemar odds ratio, 0.2; 95% CI, 0.0-0.8).

### Hospitalizations After Reinfection

A total of 426 hospitalizations occurred among 32 524 pediatric patients with COVID-19 (with primary infection) in the observed period (1.3%) (eTable 2 in Supplement 1). At the beginning of the pandemic (March to June 2020), all children with SARS-CoV-2 were hospitalized (because of official protocols in the early pandemic phase). The proportion of hospitalized patients decreased 10-fold, from 164 of 2047 (8.0%) in 2020 to 157 of 18 967 (0.8%) in 2021, and remained at a similar level in 2022 (105 of 11 510 [0.9%]). Reinfected children were rarely hospitalized: only 5 hospitalizations were recorded (0.5%), during the sixth and seventh pandemic waves. Kaplan-Meier curves for the risk of hospitalization for the entire cohort of 32 524 cases and for reinfected patients are shown in eFigure 4 in Supplement 1.

### Deaths

Three fatal outcomes were recorded among 32 524 infected children, of which 1 was classified as a COVID-19 death (case fatality ratio, 0.003%). This child, who had a serious congenital heart defect, died in February 2022. The other 2 children died of non–COVID-19 causes. All 3 deaths were recorded during the sixth pandemic wave. No deaths occurred among reinfected children and adolescents.

### Second Reinfections

Nine of 32 524 cases (0.03%) with 3 consecutive SARS-CoV-2 reinfections were recorded (eTable 3 in Supplement 1) during the sixth (n = 4) and seventh (n = 5) pandemic waves. Second reinfections occurred mostly in unvaccinated patients (8 of 9 [88.9%]) and were all mild.

## Discussion

This cohort study showed that the documented reinfection risk in children and adolescents was low before the advent of Omicron in January 2022. The risk of documented pediatric reinfection remained less than 8% at 2 years into the pandemic, which is substantially lower than the corresponding risk in the adult population. Both primary infections and reinfections in children and adolescents almost always had no clinical consequences. Severe infections and hospitalizations were rare, and only 1 child died of COVID-19. Almost all severe infections and hospitalizations and the single death occurred on primary infection. Reinfections had an even more favorable clinical course than primary infections.

A review^14^ of studies worldwide suggests that in the early pandemic phase (January to March 2020), children accounted for less than 5% of documented COVID-19 cases. Increases were noted over time, and as of April 2022, children accounted for 10% to 23% of total COVID-19 cases. This increase may largely reflect the wider use of testing with more frequent detection of asymptomatic cases, because the share of asymptomatic infections is larger in children than in adults.^15^ To some extent it may also reflect the reopening of schools and greater socialization of children as the pandemic progressed.

Data on pediatric reinfections presented to date cover mainly the pre-Omicron period. Comparisons should be made cautiously because of differences in study methods, including reinfection case definition, follow-up periods, and limitations of community testing. In 2020 to 2021, pediatric reinfections were uncommon and milder compared with primary infection.^7^ Mensah et al^6^ found an increase in reinfection rate with age. In another US-based series of pediatric reinfections until the middle of 2021, reinfections were not associated with severe or fatal outcomes.^16^ We observed an increase in the proportion of pediatric COVID-19 cases from 2.6% in 2020 to 8.9% in 2021. Testing in Serbia was limited across age groups in 2020 and became more widely available in 2021,^13^ covering a larger share of children. In 2022, pediatric COVID-19 infections in Vojvodina accounted for only 7.8%, which tends to be lower than estimates from other countries.^14^ Recent evidence suggests a robust and sustained immune response in children recovering from COVID-19, which may be related to the observed lower reinfection risk and milder clinical course in children compared with adults.^17,18^ Alternatively, this finding may herald a larger share of missed (presumably asymptomatic) reinfections in children than in adults. If so, this finding would strengthen even more the observation that pediatric reinfections have a benign clinical outlook.^1^

The incidence of reinfection in children reflected their increased exposure that accompanied trends of COVID-19 waves in the community.^6^ The explosion of reinfections caused by Omicron and its subvariants stems from the evasion of both natural and vaccine-induced immunity, often within weeks after the previous episode, thus challenging the current reinfection case definition of at least 90 days.^14,19,20,21,22^ Several studies^23,24,25^ have indicated that children infected with Omicron are less likely to experience severe disease that requires hospitalization. This finding may reflect less aggressive features of the Omicron variant and/or natural immunity from undetected prior infections.

Children and adolescents have been probably almost ubiquitously infected by the middle of 2022.^26,27,28^ In the US, a seroprevalence study^29^ suggested that 75% of children and adolescents had antibodies by February 2022. Children and adolescents seem to have exhibited largely the same infection rates as adults during the pandemic,^30^ and vaccinations may not have influenced this pattern. In Serbia, vaccination was stagnant in 2022 (47.8% of individuals completely vaccinated by June 2022),^31^ and vaccination coverage of children was negligible (<1%).

Immunization policy would benefit from additional evidence on the disease burden of emerging SARS-CoV-2 variants in healthy children and those with comorbidities, long-term vaccine efficacy trials for clinical outcomes and viral transmission, and a better understanding of post–COVID-19 condition in children. Population-based studies to monitor the reinfection rate in parallel with studies on vaccine safety among the youngest^32,33^ could help optimize vaccine policy in the future.^34,35^

### Limitations

Some limitations of our work should be discussed. First, documentation of both primary infections and reinfections was limited by the availability of testing, and unavoidably many subclinical infections were missed. Thus, the rates of clinically severe disease and complications are even lower than the extremely low rates that we observed. Given that most asymptomatic primary infections in 2020 were probably not detected, it is possible that some of the seemingly primary infections detected in 2021 were actually reinfections. It is unknown whether that group includes any substantial number of severe cases; if so, this would tend to weaken the observation that reinfections are less frequently severe than primary infections. Conversely, any missed asymptomatic reinfections after a documented severe primary infection would tend to create the opposite bias. If the proportion of missed, asymptomatic, nontested reinfections is higher in children than in adults, the lower rate of observed reinfections in children may be an artifact. Second, sequence information on infecting viruses during primary infections and reinfections could have provided valuable insights; however, sequencing was very limited at the country level during the pandemic. Third, hospitalization rates depend on the threshold for admission. All children with COVID-19 were hospitalized in the first 3 months of the pandemic based on precautionary policy choice; hospitalization rates plummeted subsequently and were close to zero for reinfections. Comparison of hospitalization data for children in other countries is precarious because it is difficult to distinguish true differences in severity from differences in admission thresholds. For example, hospitalization rates for children and adolescents with COVID-19 have been much higher in the US,^14,36^ but many admissions might have been due to diseases other than COVID-19.^37^ Many recorded pediatric COVID-19 deaths may also be due to other diseases, which may explain the higher rates of recorded COVID-19 deaths in the US compared with European countries, including Serbia.^38^ Fourth, we have not captured information on potential long-term consequences of COVID-19, although claims for frequent pediatric post–COVID-19 condition may be unfounded.^39^ Fifth, children and adolescents in the studied population were mostly unvaccinated, and our analyses were underpowered to address the impact of vaccination. However, outcomes were already excellent for pediatric infections and even more so for reinfections.

## Conclusions

This cohort study found that the SARS-CoV-2 reinfection risk remained substantially lower for children and adolescents compared with adults in Serbia as of July 2022. Pediatric infections were mild, and reinfections were even milder than primary infections. The mild nature of reinfections offers reassurance as the virus transitions to endemicity and repeated infections are to be anticipated.

## Notes

Supplement 1. **eAppendix.** The Distribution of SARS-CoV-2 Variants in Europe During the Seven Pandemic Waves Recorded in Serbia, March 6, 2020-January 31, 2022 **eTable 1.** Primary SARS-CoV-2 Infections and Reinfections in Vojvodina, Serbia, March 6, 2020-July 31, 2022 **eTable 2.** SARS-CoV-2 Primary Infections and Reinfections and Hospitalization Rates in the Pediatric Population of Vojvodina, Serbia, March 6, 2020-July 31, 2022 **eTable 3.** Characteristics of Children and Adolescents With Two Consecutive SARS-CoV-2 Reinfections in Vojvodina, Serbia, March 6, 2020-July 31, 2022 **eFigure 1.** The Proportion of Primary Infections That Were Reinfected in Children, Vojvodina, Serbia, March 6, 2020-July 31, 2022 **eFigure 2.** Kaplan-Meier Curves Showing the Cumulative Probability of Reinfection in the Pediatric Cohort (<18 Years of Age) According to (A) Severity of Primary Infection, (B) Age Group and (C) Sex in Vojvodina, Serbia, March 6, 2020-July 31, 2022 **eFigure 3.** Severity of COVID-19 in Children and Adolescents (<18 Years) Without and With SARS-CoV-2 Reinfection (First and Second Episode) in Vojvodina, Serbia, March 6, 2020-July 31, 2022 **eFigure 4.** Kaplan-Meier Curves Showing the Cumulative Probability of Hospitalization (A) in the Pediatric Cohort and (B) for Patients with Reinfection in Vojvodina, Serbia, March 6, 2020-July 31, 2022

Supplement 2.
Data Sharing Statement
