# Supplementary material for: Incidence, Risk, and Severity of SARS-CoV-2 Reinfections in Children and Adolescents Between March 2020 and July 2022 in Serbia
Source: JAMA Netw Open. 2023 Feb 13;6(2):e2255779. doi: 10.1001/jamanetworkopen.2022.55779 (PMC9926322; doi:10.1001/jamanetworkopen.2022.55779)
Supplement: Supplement 1. — eAppendix. The Distribution of SARS-CoV-2 Variants in Europe During the Seven Pandemic Waves Recorded in Serbia, March 6, 2020-January 31, 2022 eTable 1. Primary SARS-CoV-2 Infections and Reinfections in Vojvodina, Serbia, March 6, 2020-July 31, 2022 eTable 2. SARS-CoV-2 Primary Infections and Reinfections and Hospitalization Rates in the Pediatric Population of Vojvodina, Serbia, March 6, 2020-July 31, 2022 eTable 3. Characteristics of Children and Adolescents With Two Consecutive SARS-CoV-2 Reinfections in Vojvodina, Serbia, March 6, 2020-July 31, 2022 eFigure 1. The Proportion of Primary Infections That Were Reinfected in Children, Vojvodina, Serbia, March 6, 2020-July 31, 2022 eFigure 2. Kaplan-Meier Curves Showing the Cumulative Probability of Reinfection in the Pediatric Cohort (<18 Years of Age) According to (A) Severity of Primary Infection, (B) Age Group and (C) Sex in Vojvodina, Serbia, March 6, 2020-July 31, 2022 eFigure 3. Severity of COVID-19 in Children and Adolescents (<18 Years) Without and With SARS-CoV-2 Reinfection (First and Second Episode) in Vojvodina, Serbia, March 6, 2020-July 31, 2022 eFigure 4. Kaplan-Meier Curves Showing the Cumulative Probability of Hospitalization (A) in the Pediatric Cohort and (B) for Patients with Reinfection in Vojvodina, Serbia, March 6, 2020-July 31, 2022 [file jamanetwopen-e2255779-s001.pdf]

Supplementary Online Content

Medić S, Anastassopoulou C, Lozanov-Crvenković Z, et al. Incidence, risk, and severity of SARS-CoV-2 reinfections in children and adolescents between March 2020 and July 2022 in Serbia. *JAMA Netw Open*. 2023;6(2):e2255779. doi:10.1001/jamanetworkopen.2022.55779

eAppendix. The Distribution of SARS-CoV-2 Variants in Europe During the Seven Pandemic Waves Recorded in Serbia, March 6, 2020-January 31, 2022
eTable 1. Primary SARS-CoV-2 Infections and Reinfections in Vojvodina, Serbia, March 6, 2020-July 31, 2022
eTable 2. SARS-CoV-2 Primary Infections and Reinfections and Hospitalization Rates in the Pediatric Population of Vojvodina, Serbia, March 6, 2020-July 31, 2022
eTable 3. Characteristics of Children and Adolescents With Two Consecutive SARS-CoV-2 Reinfections in Vojvodina, Serbia, March 6, 2020-July 31, 2022
eFigure 1. The Proportion of Primary Infections That Were Reinfected in Children, Vojvodina, Serbia, March 6, 2020-July 31, 2022
eFigure 2. Kaplan-Meier Curves Showing the Cumulative Probability of Reinfection in the Pediatric Cohort (<18 Years of Age) According to (A) Severity of Primary Infection, (B) Age Group and (C) Sex in Vojvodina, Serbia, March 6, 2020-July 31, 2022
eFigure 3. Severity of COVID-19 in Children and Adolescents (<18 Years) Without and With SARS-CoV-2 Reinfection (First and Second Episode) in Vojvodina, Serbia, March 6, 2020-July 31, 2022
eFigure 4. Kaplan-Meier Curves Showing the Cumulative Probability of Hospitalization (A) in the Pediatric Cohort and (B) for Patients With Reinfection in Vojvodina, Serbia, March 6, 2020-July 31, 2022

This supplementary material has been provided by the authors to give readers additional information about their work.

© 2023 Medić S et al. *JAMA Network Open.*

eAppendix. The Distribution of SARS-CoV-2 Variants in Europe During the Seven Pandemic Waves Recorded in Serbia, March 6, 2020-January 31, 2022. (Nextstrain naming was used).

1. First pandemic wave (Mar. 6 - Jun. 1, 2020): 19A, 19B, 20A, 20B, 20C, 20D.
2. Second pandemic wave (Jun. 2 - Oct. 6, 2020): 19B, 20A, 20B, 20C, 20D, 20E (EU1).
3. Third pandemic wave (Oct. 7, 2020 - Jan. 31, 2021): 19B, 20A, 20B, 20C, 20D, 20E (EU1), 20H (Beta, V2), 20I (Alpha, V1), 21C (Epsilon), 21D (Eta).
4. Fourth pandemic wave (Feb. 1 - Jul. 23, 2021): 19B, 20A, 20B, 20C, 20D, 20E (EU1), 20H (Beta, V2), 20I (Alpha, V1), 20J (Gamma, V3), 21A (Delta), 21I (Delta), 21J (Delta), 21B (Kappa), 21D (Eta), 21G (Lambda).
5. Fifth pandemic wave (Jul. 24 - Dec. 31, 2021): 19B, 20A, 20B, 20C, 20E (EU1), 20I (Alpha, V1), 20J (Gamma, V3), 21A (Delta), 21I (Delta), 21J (Delta), 21H (Mu), 21K (Omicron), 21L (Omicron).
6. Sixth pandemic wave (Jan. 1 - Jun. 30, 2022): 21I (Delta), 21J (Delta), 21K (Omicron), 21L (Omicron).
7. Seventh (ongoing) pandemic wave (Jul. 1, 2022 -): 21L (Omicron), 22A (Omicron), 22B (Omicron).

© 2023 Medić S et al. *JAMA Network Open.*

eTable 1. Primary SARS-CoV-2 Infections and Reinfections in Vojvodina, Serbia, March 6, 2020-July 31, 2022.

	Months	Overall COVID-19 cases[a]		SARS-CoV-2 reinfections[b]		Pediatric COVID-19 cases[a,c]		Reinfections in the pediatric population[b,c]		Share of pediatric cases in the total number of COVID-19 cases	Share of SARS-CoV-2 reinfections in children in the total number of reinfections
		**n**	**%**	**n**	**%**	**n**	**%**	**n**	**%**	**%**	**%**
**2020**	**March-June**	1570	0.4	0	0	68	0.2	0	0	4.3	0
	**July**	4597	1.0	0	0	83	0.3	0	0	1.8	0
	**August**	1750	0.4	0	0	33	0.1	0	0	1.9	0
	**September**	330	0.1	0	0	8	0.02	0	0	2.4	0
	**October**	1547	0.3	1	0.06	29	0.1	0	0	1.9	0
	**November**	30 659	6.7	9	0.03	854	2.5	0	0	2.8	0
	**December**	38 392	8.4	15	0.04	972	2.9	0	0	2.5	0
	**2020 subtotal**	**78 845**	**17.3**	**25**	**0.03**	**2047**	**6.1**	**0**	**0**	**2.6**	**0**
**2021**	**January**	11 441	2.5	7	0.06	357	1.1	0	0	3.1	0
	**February**	11 868	2.6	11	0.09	459	1.4	0	0	3.9	0
	**March**	30 012	6.6	47	0.2	1201	3.6	0	0	4.0	0
	**April**	21 929	4.8	47	0.2	983	2.9	1	0.1	4.5	2.1
	**May**	5084	1.1	21	0.4	285	0.8	1	0.4	5.6	4.8
	**June**	586	0.1	5	0.9	55	0.2	0	0	9.4	0
	**July**	860	0.2	8	0.9	47	0.1	0	0	5.5	0
	**August**	7029	1.5	80	1.1	544	1.6	1	0.2	7.7	1.3
	**September**	35 515	7.8	351	1.0	5015	14.8	14	0.3	14.1	4.0
	**October**	47 935	10.5	567	1.2	6506	19.3	24	0.4	13.6	4.2

© 2023 Medić S et al. *JAMA Network Open.*

	Months	Overall COVID-19 cases[a]		SARS-CoV-2 reinfections[b]		Pediatric COVID-19 cases[a,c]		Reinfections in the pediatric population[b,c]		Share of pediatric cases in the total number of COVID-19 cases	Share of SARS-CoV-2 reinfections in children in the total number of reinfections
		**n**	**%**	**n**	**%**	**n**	**%**	**n**	**%**	**%**	**%**
	**November**	32 174	7.1	526	1.6	2798	8.3	11	0.4	8.7	2.1
	**December**	9212	2.0	186	2.0	717	2.1	7	1.0	7.8	3.8
	**2021 subtotal**	**213 645**	**46.8**	**1856**	**0.9**	**18 967**	**56.1**	**59**	**0.3**	**8.9**	**3.2**
**2022**	**January**	82 046	18.0	11 967	14.6	7638	22.6	420	5.5	9.3	3.5
	**February**	45 713	10.0	5535	12.1	3261	9.6	225	6.9	7.1	4.1
	**March**	9710	2.1	1257	12.9	500	1.5	69	13.8	5.2	5.5
	**April**	4562	1.0	639	14.0	111	0.3	9	8.1	2.4	1.4
	**May**	1829	0.4	263	14.4	70	0.2	8	11.4	3.8	3.0
	**June**	1924	0.4	358	18.6	76	0.2	6	7.9	3.9	1.7
	**July**	18 060	4.0	4116	22.8	1112	3.3	168	15.1	6.2	4.1
	**2022 subtotal**	**163 844**	**35.9**	**24 135**	**14.7**	**12 768**	**37.8**	**905**	**7.1**	**7.8**	**3.8**
**Total 2020-2022**		**456 334**	**100**	**26 016**	**5.7**	**33 782**	**100**	**964**	**2.9**	**7.4**	**3.7**

[a] By month of first episode registration.
[b] By month of reinfection registration.
[c] Patients aged <18 years.

© 2023 Medić S et al. *JAMA Network Open.*

eTable 2. SARS-CoV-2 Primary Infections and Reinfections and Hospitalization Rates in the Pediatric Population[a] of Vojvodina, Serbia, March 6, 2020-July 31, 2022.

		Pediatric SARS-CoV-2 primary infections[b]		Hospitalized primary infections[b, c]		Proportion of SARS-CoV-2 reinfections in the total number of reinfections		Proportion of SARS-CoV-2 reinfections in the number of primary infections[d]		Proportion of hospitalized reinfections[c,d]		Primary SARS-CoV-2 infections that were reinfected	
	**Months**	**n**	**%**	**n**	**%**	**n**	**%**	**n**	**%**	**n**	**%**	**n**	**%**
**2020**	March-June	68	0.2	68	100.0	0	0	0	0	0	0	0	0
	July	83	0.3	45	54.2	0	0	0	0	0	0	2	2.4
	August	33	0.1	6	18.2	0	0	0	0	0	0	1	3.0
	September	8	0.02	1	12.5	0	0	0	0	0	0	0	0
	October	29	0.1	1	3.5	0	0	0	0	0	0	0	0
	November	854	2.6	21	2.5	0	0	0	0	0	0	65	7.6
	December	972	3.0	22	2.3	0	0	0	0	0	0	75	7.7
	**Subtotal**	**2047**	**6.3**	**164**	**8.0**	**0**	**0**	**0**	**0**	**0**	**0**	**143**	**7.0**
**2021**	January	357	1.1	4	1.1	0	0	0	0	0	0	33	9.2
	February	459	1.4	5	1.1	0	0	0	0	0	0	35	7.6
	March	1201	3.7	19	1.6	0	0	0	0	0	0	100	8.3
	April	983	3.0	17	1.7	1	0.1	1	0.1	0	0	79	8.0
	May	285	0.9	5	1.8	1	0.1	1	0.4	0	0	19	6.7
	June	55	0.2	3	5.5	0	0	0	0	0	0	7	12.7
	July	47	0.2	3	6.4	0	0	0	0	0	0	3	6.4
	August	544	1.7	6	1.1	1	0.1	1	0.2	0	0	23	4.2
	September	5015	15.4	24	0.5	14	1.5	14	0.3	0	0	175	3.5
	October	6506	20.0	31	0.5	24	2.5	24	0.4	0	0	227	3.5
	November	2798	8.6	27	1.0	11	1.2	11	0.4	0	0	53	1.9
	December	717	2.2	13	1.8	7	0.7	7	1.0	0	0	12	1.7
	**Subtotal**	**18 967**	**58.3**	**157**	**0.8**	**59**	**6.1**	**59**	**0.3**	0	**0**	**766**	**4.0**
**2022**	January	7638	23.5	37	0.5	420	43.6	420	5.5	4	1.0	42	0.6
	February	3261	10.0	46	1.4	225	23.3	225	6.9	0	0	11	0.3

© 2023 Medić S et al. *JAMA Network Open.*

		Pediatric SARS-CoV-2 primary infections[b]		Hospitalized primary infections[b, c]		Proportion of SARS-CoV-2 reinfections in the total number of reinfections		Proportion of SARS-CoV-2 reinfections in the number of primary infections[d]		Proportion of hospitalized reinfections[c,d]		Primary SARS-CoV-2 infections that were reinfected	
	**Months**	**n**	**%**	**n**	**%**	**n**	**%**	**n**	**%**	**n**	**%**	**n**	**%**
	March	500	1.5	16	3.2	69	7.2	69	13.8	0	0	2	0.4
	April	111	0.3	6	5.4	9	0.9	9	8.1	0	0	0	0
	May	-	-	-	-	8	0.8	8	-	0	0	-	-
	June	-	-	-	-	6	0.6	6	-	0	0	-	-
	July	-	-	-	-	168	17.4	168	-	1	0.6	-	-
	**Subtotal**	**11 510**	**35.4**	**105**	**0.9**	**905**	**93.9**	**905**	**7.9**	**5**	**0.6**	**55**	**0.5**
**TOTAL**		**32 524**	**100**	**426**	**1.3**	**964**	**100**	**964**	**3.0**	**5**	**0.5**	**964**	**3.0**

[a] Aged <18 years.
[b] By month of first episode registration.
[c] Hospitalization within one month from the date of laboratory confirmation of infection or reinfection.
[d] By month of reinfection registration.

© 2023 Medić S et al. *JAMA Network Open.*

eTable 3. Characteristics of Children and Adolescents[a] With Two Consecutive SARS-CoV-2 Reinfections in Vojvodina, Serbia, March 6, 2020-July 31, 2022.

Sex	Age[b] (years)	Number of comorbidities[b]	Vaccination status[b]	Time between infections (days)		Pandemic wave[d]		
				1st infection to 1st reinfection	1st reinfection to 2nd reinfection	Primary infection[c]	1st reinfection[c]	2nd reinfection[c]
Male	10	0	Unvaccinated	224	163	4	5	7
Male	11	0	Unvaccinated	131	129	5	6	6
Male	13	0	Unvaccinated	125	134	5	6	6
Male[e]	16	1	Unvaccinated	494	132	3	6	7
Female	3	0	Unvaccinated	167	188	5	6	7
Female	13	0	Unvaccinated	121	135	5	6	7
Female	13	0	Unvaccinated	308	120	3	5	6
Female	14	0	Unvaccinated	149	164	5	6	7
Female	17	0	Partially vaccinated[f]	208	319	4	5	6

[a] Aged <18 years.
[b] At the date of laboratory confirmation of second SARS-CoV-2 reinfection.
[c] At the date of SARS-CoV-2 laboratory confirmation of SARS-CoV-2 infection.
[d] Time duration of pandemic waves: Second wave: Jun. 2-Oct. 6, 2020; Third wave: Oct. 7, 2020-Jan. 31, 2021; Fourth wave: Feb. 1-Jul. 23, 2021; Fifth wave: Jul. 24-Dec. 31, 2021; Sixth wave: Jan.-Feb. 2022; Seventh (ongoing) wave: Jul. 1, 2022-.
[e] The 16-year-old male with consecutive reinfections was obese.
[f] Vaccinated with one dose of BNT162b2, 49 days before symptom onset of second SARS-CoV-2 reinfection.

© 2023 Medić S et al. *JAMA Network Open.*

eFigure 1. The Proportion of Primary SARS-CoV-2 Infections That Were Reinfected in Children, Vojvodina, Serbia, March 6, 2020-July 31, 2022.

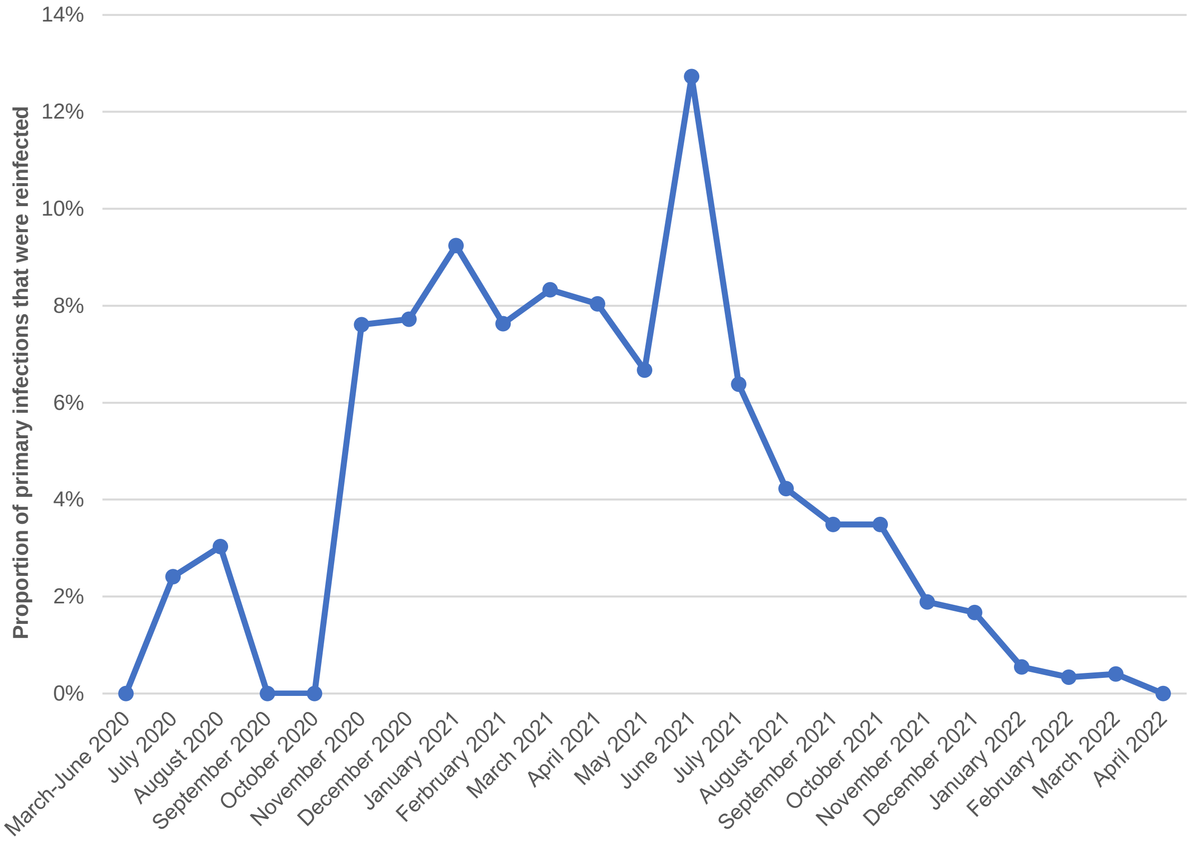

© 2023 Medić S et al. *JAMA Network Open.*

eFigure 2. Kaplan-Meier Curves Showing the Cumulative Probability of Reinfection in the Pediatric Cohort (<18 Years of Age) According to (A) Severity of Primary Infection, (B) Age Group and (C) Sex in Vojvodina, Serbia, March 6, 2020-July 31, 2022.

A

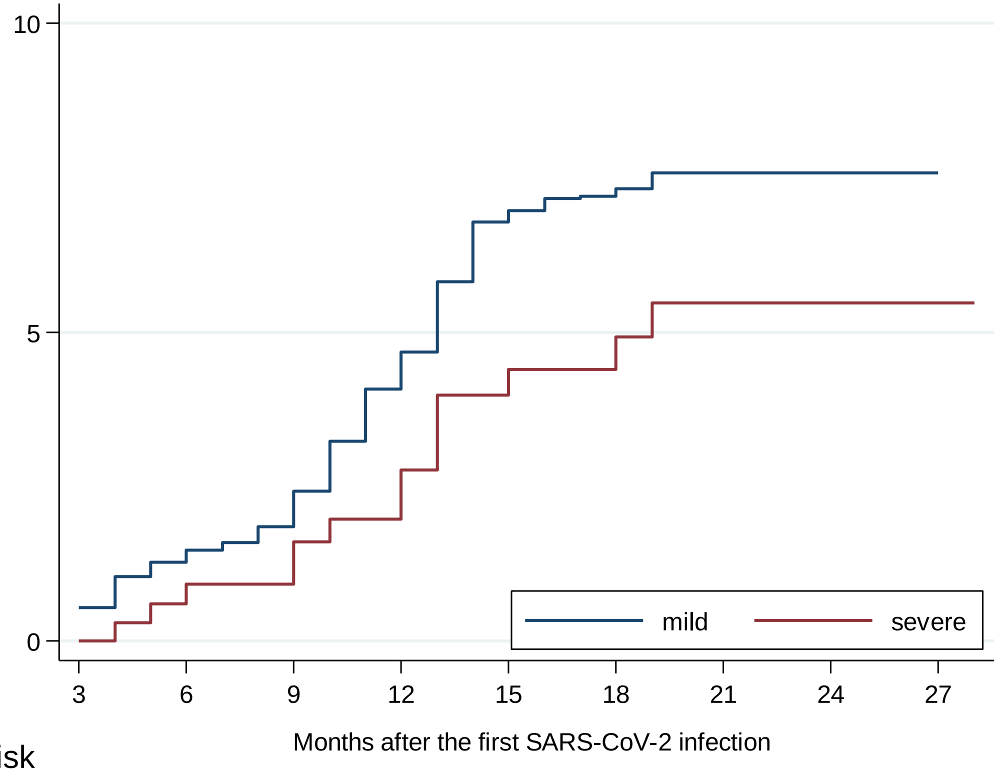

Number at risk									
mild	32182	28371	17464	4960	4562	2146	166	44	1
severe	339	316	288	246	233	180	125	115	44

© 2023 Medić S et al. *JAMA Network Open.*

B

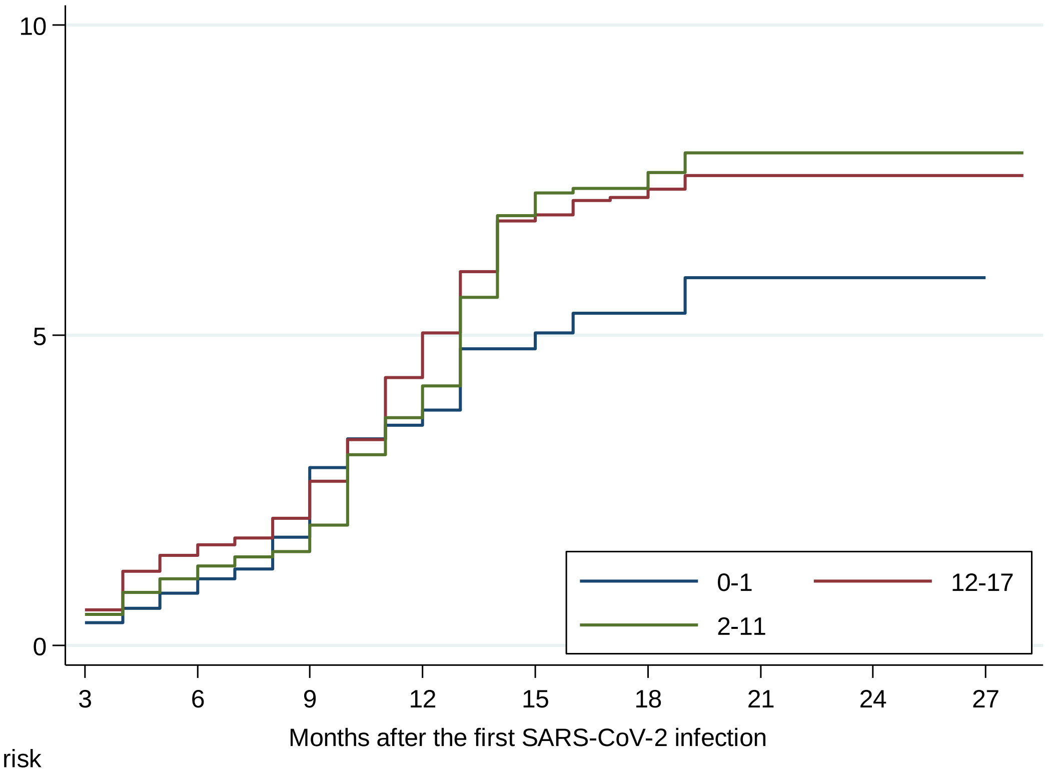

Number at risk

0-1	2171	1749	956	396	368	193	26	17	2
2-11	11918	10226	5298	1707	1562	699	100	63	25
12-17	18432	16712	11498	3103	2865	1434	165	79	18

© 2023 Medić S et al. *JAMA Network Open.*

C

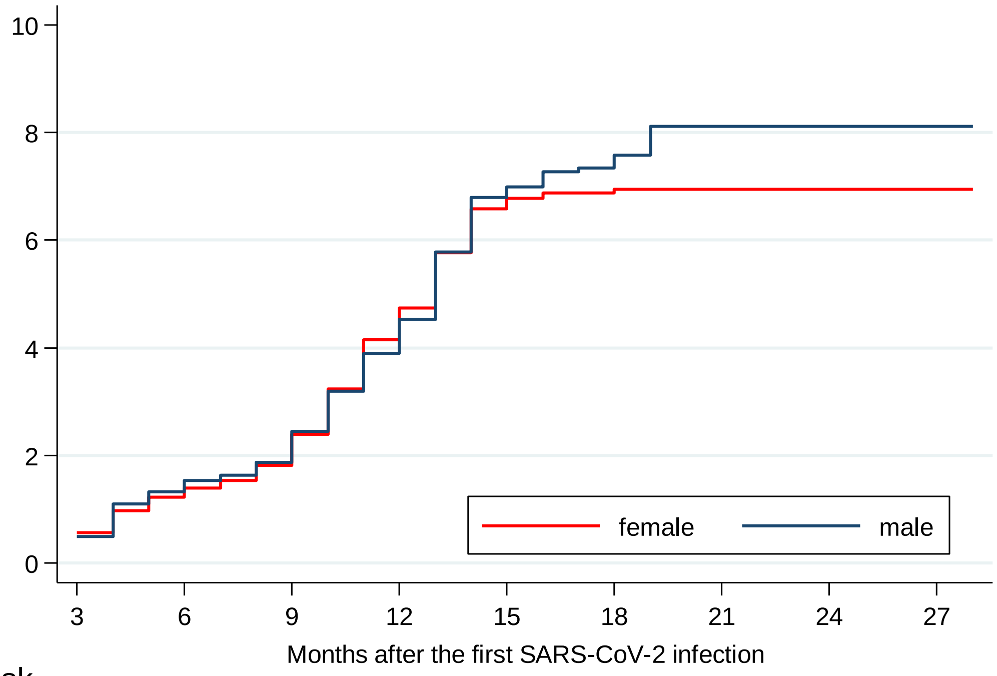

Number at risk									
female	15951	14154	8880	2630	2429	1149	126	67	21
male	16570	14533	8872	2576	2366	1177	165	92	24

© 2023 Medić S et al. *JAMA Network Open.*

eFigure 3. Severity of COVID-19 in Children and Adolescents (<18 years) Without and With SARS-CoV-2 Reinfection (First and Second Episode) in Vojvodina, Serbia, March 6, 2020-July 31, 2022.

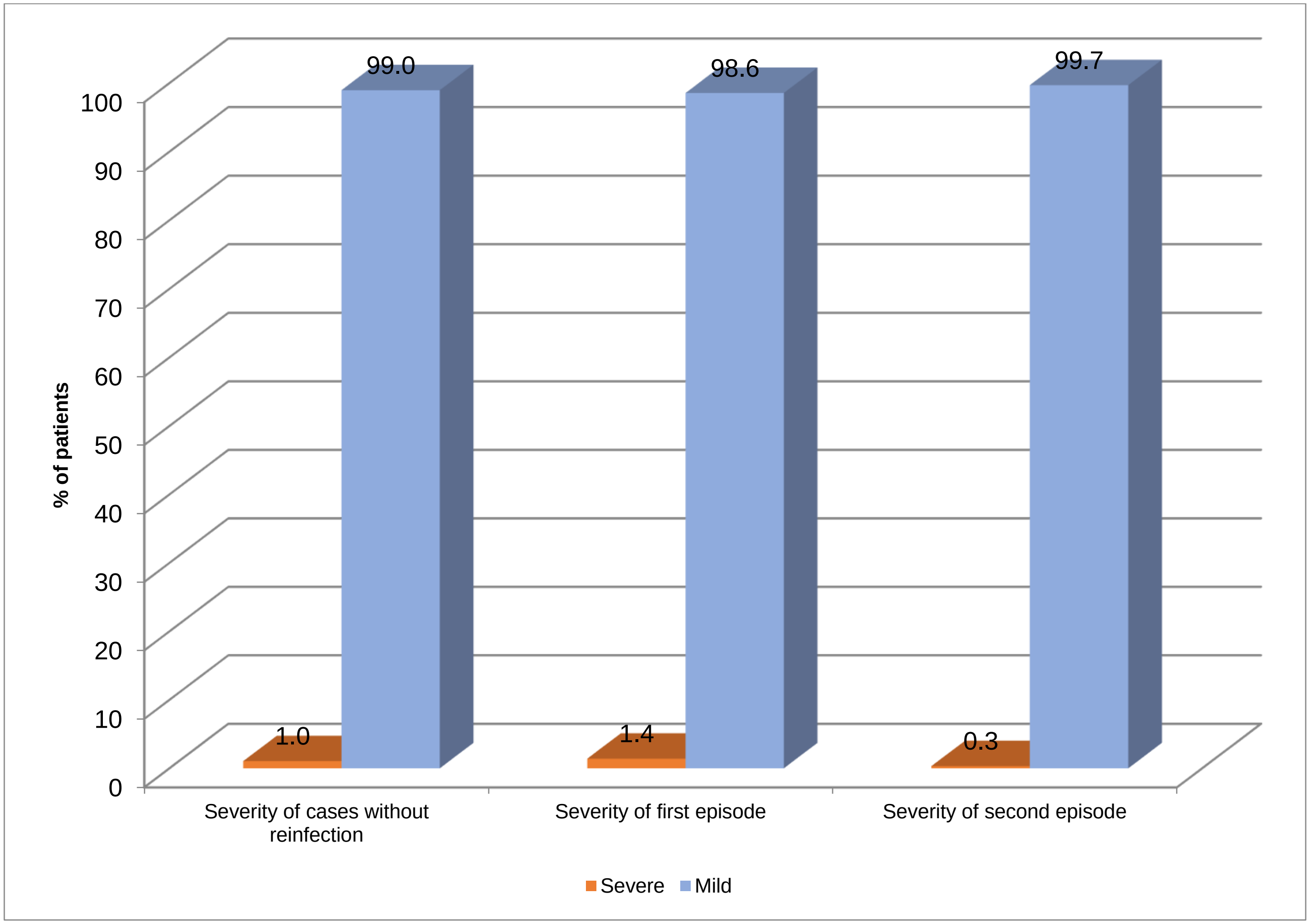

© 2023 Medić S et al. *JAMA Network Open.*

eFigure 4. Kaplan-Meier Curves Showing the Cumulative Probability of Hospitalization (A) in the Pediatric Cohort and (B) for Patients With Reinfection in Vojvodina, Serbia, March 6, 2020-July 31, 2022.

A

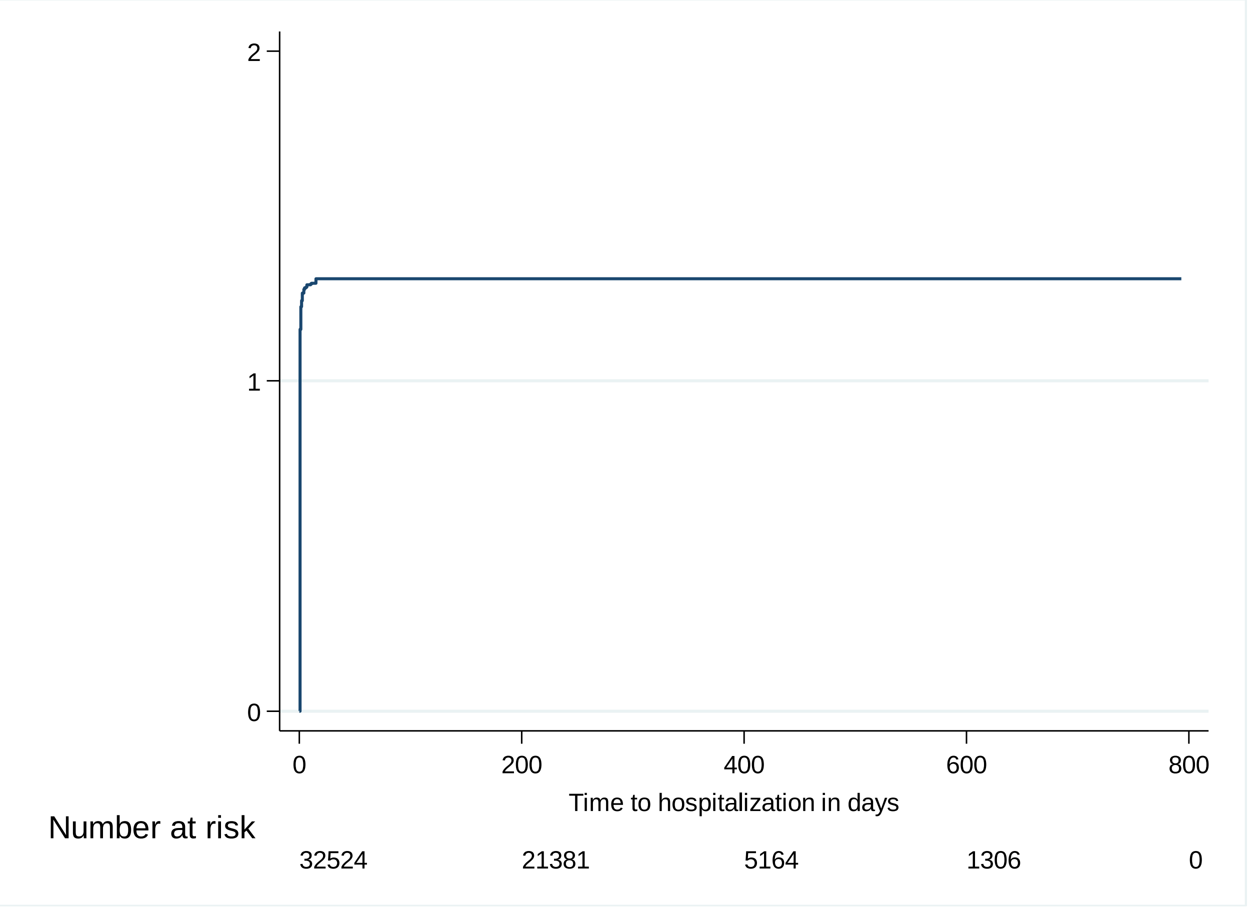

© 2023 Medić S et al. *JAMA Network Open.*

B

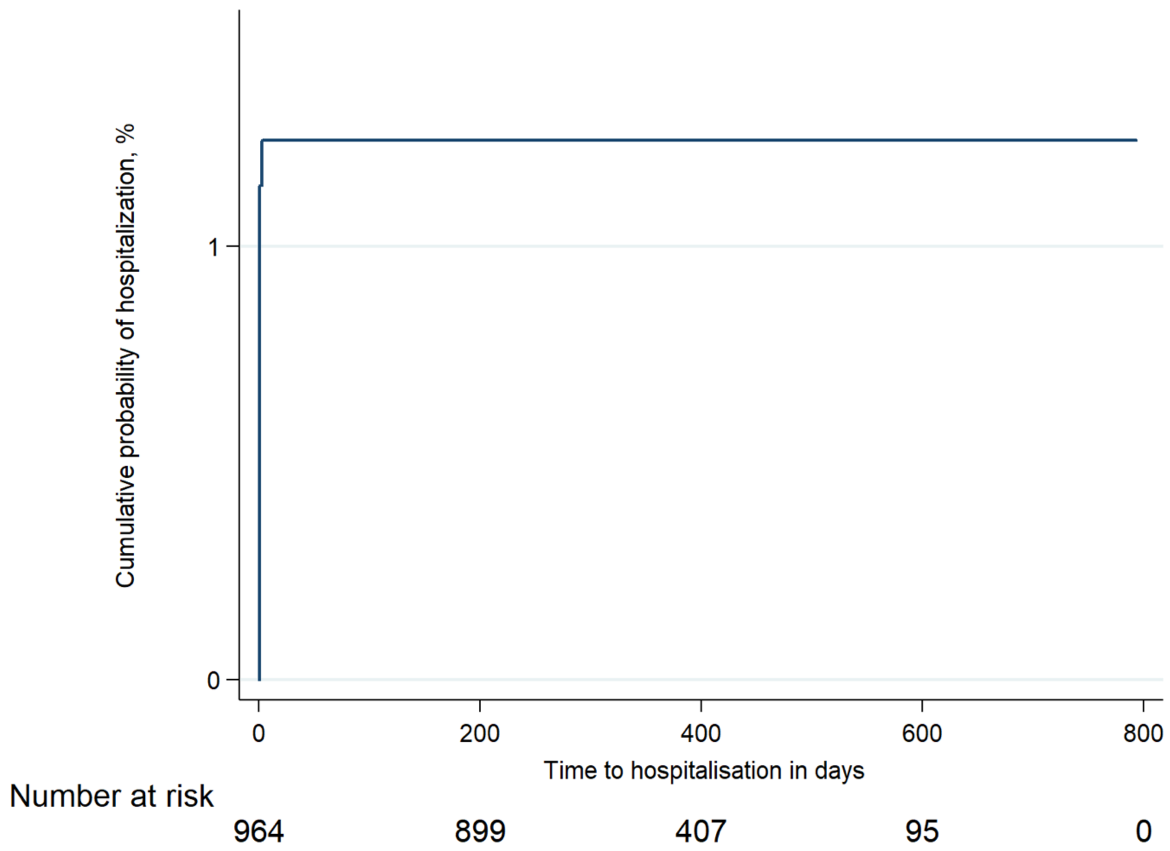

© 2023 Medić S et al. *JAMA Network Open.*
