# Supplementary material for: Incidence, Risk, and Severity of SARS-CoV-2 Reinfections in Children and Adolescents Between March 2020 and July 2022 in Serbia
Source: JAMA Netw Open. 2023 Feb 13;6(2):e2255779. doi: 10.1001/jamanetworkopen.2022.55779 (PMC9926322; doi:10.1001/jamanetworkopen.2022.55779)
Supplement: Supplement 2. — Data Sharing Statement [file jamanetwopen-e2255779-s002.pdf]

Data Sharing Statement

Medić. Incidence, Risk, and Severity of SARS-CoV-2 Reinfections in Children and Adolescents Between March 2020 and July 2022 in Serbia. *JAMA Netw Open*. Published February 13, 2023. doi:10.1001/jamanetworkopen.2022.55779

Data
Data available: No

Additional Information
Explanation for why data not available: Some data may be available after communication with the primary investigator and approval by the Voivodina authorities.
